# Bilirubin is an Endogenous Antioxidant in Human Vascular Endothelial Cells

**DOI:** 10.1038/srep29240

**Published:** 2016-07-06

**Authors:** Lovro Ziberna, Mitja Martelanc, Mladen Franko, Sabina Passamonti

**Affiliations:** 1 University of Trieste, Department of Life Sciences, via L. Giorgeri 1, 34127 Trieste, Italy; 2 Univesity of Ljubljana, Faculty of Medicine, Institute of Pharmacology and Experimental Toxicology, Korytkova 2, 1000 Ljubjana, Slovenia; 3 University of Nova Gorica, The Laboratory for Environmental Research, Vipavska 13, POB 301, 5001 Nova Gorica, Slovenia

## Abstract

Bilirubin is a standard serum biomarker of liver function. Inexplicably, it is inversely correlated with cardiovascular disease risk. Given the role of endothelial dysfunction in originating cardiovascular diseases, direct analysis of bilirubin in the vascular endothelium would shed light on these relationships. Hence, we used high-performance liquid chromatography coupled with thermal lens spectrometric detection and diode array detection for the determination of endogenous cellular IXα-bilirubin. To confirm the isomer IXα-bilirubin, we used ultra-performance liquid chromatography coupled with a high-resolution mass spectrometer using an electrospray ionization source, as well as tandem mass spectrometric detection. We measured bilirubin in both arterial and venous rat endothelium (0.9–1.5 pmol mg^−1^ protein). In the human endothelial Ea.hy926 cell line, we demonstrated that intracellular bilirubin (3–5 pmol mg^−1^ protein) could be modulated by either extracellular bilirubin uptake, or by up-regulation of heme oxygenase-1, a cellular enzyme related to endogenous bilirubin synthesis. Moreover, we determined intracellular antioxidant activity by bilirubin, with EC50 = 11.4 ± 0.2 nM, in the range of reported values of free serum bilirubin (8.5–13.1 nM). Biliverdin showed similar antioxidant properties as bilirubin. We infer from these observations that intra-endothelial bilirubin oscillates, and may thus be a dynamic factor of the endothelial function.

Bilirubin is a tetrapyrrole pigment present in various chemical forms in the blood, namely, conjugated with glucuronic acid (direct bilirubin), unconjugated bound to serum albumin (indirect bilirubin) and unconjugated-unbound (free bilirubin)^1^. Bilirubin is formed in cells by two sequential reactions, catalysed by heme oxygenase (biliverdin-producing, EC 1.14.99.3) and biliverdin reductase (EC 1.3.1.24). Approximately 250–300 mg bilirubin/day is formed in a normal adult. Most of bilirubin arises in the spleen from the catabolism of haemoglobin released form red blood cells. However, the pathway of heme catabolism is active in any kind of cells, supporting the turnover of heme, the cofactor of cytochromes and many other enzymes.

Bilirubin has antioxidant and anti-inflammatory activity, and is inversely correlated with disease risk of the cardiovascular system, such as ischemic heart disease, hypertension, diabetes type II, metabolic syndrome, and obesity, among others^2^,^3^. The vascular endothelium that lines the blood vessels plays a fundamental role in cardiovascular disease onset and progression^4^. Recent studies on the cellular composition of human and mouse tissue showed that endothelial cells are the most abundant cells in the heart—about 60 percent – thus implying to have an important role in heart physiology and pathology^5^. However, it is not known if the vascular endothelium is the transducer of the ‘bilirubin factor’ put in evidence by the aforementioned epidemiological studies. Therefore, the aim of our study was to provide the first ever, direct experimental proof for the presence and bioactivity of bilirubin in the vascular endothelium.

## Results and Discussion

### Is bilirubin an endothelial protective agent?

**Figure 1:**
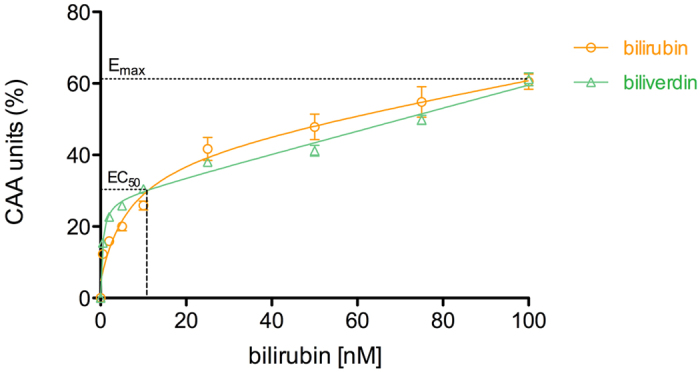
Endothelial cellular antioxidant activity (CAA) of bilirubin, and its synthetic precursor biliverdin. Endothelial cells (Ea.hy926) were exposed to different concentrations of bilirubin or biliverdin (0.5, 2, 5, 10, 25, 50, 75 and 100 nM) in the albumin-free cell medium for a period of 30 min before the start of CAA assay. Results are expressed as means ± SEM from hexaplicates of 3 independent experiments.

To assess the importance of bilirubin in endothelial protection from oxidative stress, we used the standard cell line EA.hy926 as a model of the human vascular endothelium. Cells were challenged with an oxidative stress, detected as a time-dependent increase of fluorescence emission by an intracellular probe. We assayed the effect of low concentrations of bilirubin (in the nM range) for 30 min, finding a concentration-dependent decrease in fluorescence. Data were plotted to quantify the antioxidant effect (Fig. 1) and obtain the EC50. The value found (11.4 ± 0.2 nM) was in the range of reported values of free serum bilirubin (8.5–13.1 nM)^1^. In such a range, the observed system is the most adaptable to changes (highest slope). This finding means that small changes in the extracellular bilirubin concentrations can be translated into substantially improved intracellular defence against oxidative stress. Biliverdin showed similar antioxidant properties as bilirubin, likely due to its conversion to bilirubin by biliverdin reductase^6^.

### Is endothelial bilirubin measurable?

Based on these findings, we attempted at analysing intra-cellular free bilirubin, by an analytical HPLC method that replaced the standard diode array detector (DAD) detector with the ultra-highly sensitive thermal lens spectrometric detection (TLS). The quantification of intracellular bilirubin has never been attempted before, due to sensitivity limits of the current analytical methods. Only recently has a breakthrough in free bilirubin direct assessment in human and animal serum been accomplished, using a hyphenated HPLC-TLS technique^1^,^7^. The application of TLS detection instead of DAD has lowered the limits of both detection (LOD) and quantification (LOQ) by as much as 20-fold^1^.

**Figure 2:**
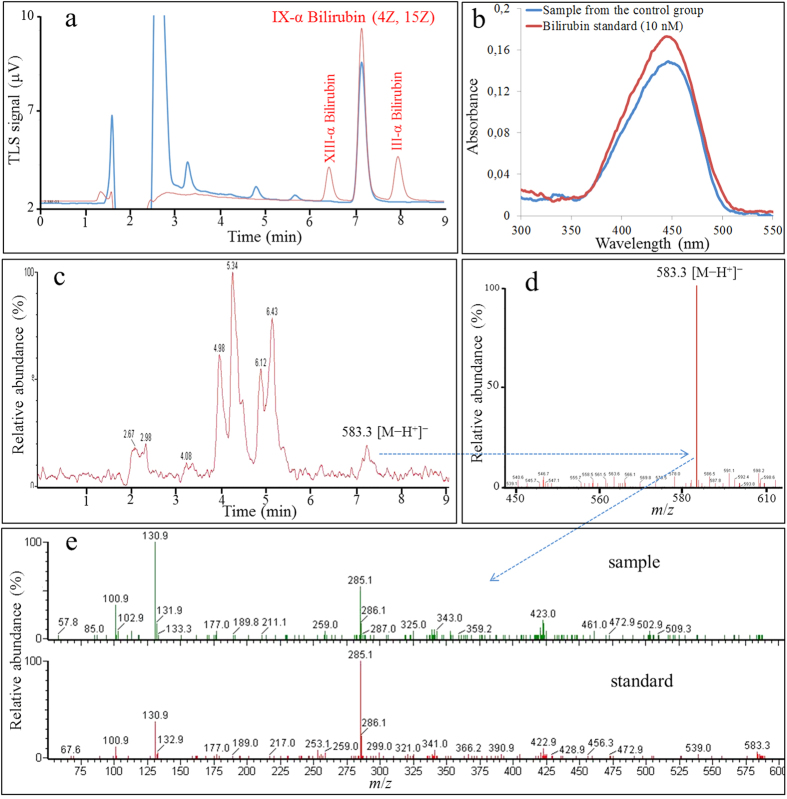
Bilirubin determination in endothelial cells. Endogenous bilirubin in endothelial cells (Ea.hy926) was identified by HPLC-TLS, HPLC-DAD or UPLC-ESI-MS-MS system. (**a**) HPLC-TLS chromatogram of bilirubin standard solution containing the three bilirubin isomers (III, IX, XIII - red line) and the endothelial cellular fraction (blue line). (**b**) HPLC-DAD chromatograms of bilirubin standard (10 nM, red line) and endothelial cellular fraction (blue line). (**c**) UPLC-ESI-MS chromatogram of analysed endothelial cellular fraction with peaks representing ions with *m*/*z* value of 583.3 [M−H^+^]^−^. (**d**) MS spectrum containing ion with *m*/*z* value of 583.3 [M−H^+^]^−^ obtained after UPLC-ESI-MS analyses at retention time of bilirubin standard elution. (**e**) MS-MS spectrum containing ion with *m*/*z* value of 285.1 after UPLC-ESI-MS-MS of bilirubin standard and the sample of endothelial cellular fraction.

Figure 2 shows the analysis of endogenous bilirubin in endothelial cells, based on MS and MS-MS fragmentation patterns, high-resolution mass (presented in Supplementary information), and retention time using HPLC-TLS, HPLC-DAD or UPLC-ESI-MS-MS system. Firstly, we used HPLC-TLS, due to its ultra-high sensitivity. Figure 2a shows the HPLC-TLS chromatogram of bilirubin standard solution containing three bilirubin isomers (III, IX, XIII-red line) and the endothelial cellular fraction (blue line). Co-elution of both HPLC peaks proved the presence of IXα bilirubin in the sample, devoid of unnaturally occurring III and XIII bilirubin isomers. Then, we applied an HPLC-DAD method, which enabled us to successfully perform the peak purity test by determining spectral homogeneity across the chromatographic peak, despite the fact that bilirubin concentration levels were at the LOQ (see Figure S1 in Supplementary information). The absorbance spectra obtained in both the region of eluted peak of standard bilirubin and in the peak present in the sample showed identical absorption pattern (Fig. 2b). Figure 2c shows UPLC-ESI-MS chromatogram of the endothelial cellular fraction, with peaks representing ions with *m*/*z* value of 583.3 [M−H^+^]^−^. This value was the same as that seen with bilirubin standard (Fig. 2d). Figure 2e shows that UPLC-ESI-MS-MS additionally confirms the presence of intracellular bilirubin through comparison between the ion fragments pattern in the MS-MS spectra of bilirubin standard and the endothelial cellular fraction, both obtained after collision-induced fragmentation of pseudo-molecular ion (583.3 [M−H^+^]^−^); they showed an identical fragmentation pattern containing a typical bilirubin ion fragment corresponding to the *m*/*z* value of 285.1.

The detector coupled to the HPLC system, i.e. either one based on TLS or a DAD, determines the performance of the methods. The HPLC-DAD method is limited by its sensitivity, which results in higher LOQ (3.5 pmol mg^−1^ protein) compared to HPLC-TLS (LOQ = 0.12 pmol mg^−1^ protein), and consequently in higher measurement error and therefore in lower accuracy of the results. For example, the LOQ of HPLC-DAD is about the same as changes in intracellular bilirubin levels observed in this study (3–5 pmol mg^−1^ protein).

In addition to endothelial cell culture experiments (EA.hy926 cells), we confirmed the presence of endogenous bilirubin in the endothelium isolated from both rat aorta and vena cava inferior. The values obtained were 1.53 ± 0.57 and 0.93 ± 0.14 pmol bilirubin mg^−1^ proteins (means ± SEM, n = 3), respectively.

### Is endothelial bilirubin dynamically regulated?

The remarkable gain in sensitivity offered by combining HPLC with TLS detection enabled to address the question whether the vascular endothelium is a bilirubin-accessible compartment. Many clinical studies have shown the potential to modulate serum bilirubin values by either lifestyle modifications or pharmacological interventions targeting bilirubin transporters and enzymes, e.g. biliverdin reductase, heme oxygenase, UDP-glucuronosyltransferase (UGT1A1), organic anion-transporting polypeptide (OATP), and others. In turn, these changes can have an impact on individual cardiovascular disease risk^8^, as well as can be used as an early biomarker for the development of metabolic syndrome^9^. However, it is so far unanswered if bilirubin contents in the vascular endothelium can be modulated, which could result in a different capacity to withstand oxidative stress and limit cellular structural damage.

Thus, we designed two experiments aimed to assess dynamic changes in cellular bilirubin levels. In the first one, EA.hy926 cells were treated with 10 μM CoPPIX for 24 hours to induce the enzyme heme oxygenase-1 and thus increase cellular bilirubin synthesis. In the second one, the cells were kept in albumin-free cell media supplemented with 50 nM bilirubin for 30 min. The HPLC-TLS method enabled to measure increased intracellular bilirubin in both cases. Specifically, it was 3.14 ± 0.17, 4.34 ± 0.26 and 5.24 ± 0.51 pmol mg^−1^ protein (means ± SEM from triplicates of 6 independent experiments) under control conditions, heme oxygenase induction (P > 0.05) and bilirubin supplementation (P > 0.001), respectively. Though other technologies are needed to study the exact localization of bilirubin in the cells, it can be guessed that bilirubin can be present in: i) the cytosol, as either free or bound to intracellular proteins (e.g. glutathione-S-transferase A, previously known as ligandin); ii) cell membranes or iii) intracellular membrane compartments, due to its lipophilic character^10^. Whatever the case may be, these data corroborate the concentration-dependent antioxidant activity of bilirubin (shown in Fig. 1), either as a lipid peroxidation inhibitor or as a radical scavenger^11^,^12^.

## Conclusions

Our data explain why increased HO-1 expression level can lead to improved disease outcomes^13^. Moreover, this study enables to conclude that the protection against cardiovascular disease risk afforded by familial hyperbilirubinemia (Gilbert’s syndrome)^14^ is indeed mediated by an increased availability of the intracellular biliverdin/bilirubin redox pair, which improves the performance of the vascular endothelium in buffering reactive oxygen and nitrogen species^15^.

Given its anatomical size (ca. 240 m^2^ in a human subject)^16^, the contribution of the vascular endothelium to the physiological redox homeostasis is most prominent.

## Materials and Methods

### Chemicals

Methanol and anhydrous dimethyl sulfoxide (DMSO), both HPLC grade, were obtained from Merck (Darmstadt, Germany). Fetal bovine serum (FBS), L-glutamine and penicillin-streptomycin solution were obtained from EuroClone (Milano, Italy); and 2′,7′-dichlorofluorescin diacetate (DCFH-DA), 2,2′-azobis (2-amidinopropane) dihydrochloride (ABAP), cobalt-protoporphyrin IX (CoPPIX), bilirubin, and biliverdin were purchased from Sigma-Aldrich (Steinheim, Germany).

Phosphate Saline buffer (PBS): 200 mg/L KCl (Merck, Darmstadt, Germany); 200 mg/L KH_2_PO_4_ (Carlo Erba, Milano, Italy); 8000 mg/L NaCl; 1150 mg/L Na_2_HPO_4_ (Carlo Erba, Milan, Italy). Hank’s buffered saline solution (HBSS): 185 mg/L CaCl_2_ × 2H_2_O; 60 mg/L KH_2_PO_4_; 350 mg/L NaHCO_3_; 8000 mg/L NaCl; 47.88 mg/L Na_2_HPO_4_ (Carlo Erba, Milano, Italy); 100 mg/L MgCl_2_ × 6H_2_O; 1000 mg/L glucose (Sigma-Aldrich, Steinheim, Germany); 100 mg/L MgSO_4_ × 7H_2_O; 400 mg/L KCl (Merck, Darmstadt, Germany); the pH was adjusted to 7.4 using 0.1% HCl (Merck, Darmstadt, Germany). Ultrapure Milli-Q water (Merck Millipore, Billerica, MA) was used for the preparation of the solutions.

### Bilirubin solutions

Bilirubin stock solution (5 mM) was prepared in DMSO. Then, it was diluted to 100 μM in 10 mM NaOH aqueous solution. Further diluted solutions were prepared using PBS. All dilutions were done in a dark room to avoid bilirubin photodegradation.

### Cell culture

Human endothelial cell line Ea.hy926 (American Type Culture Collection, Rockville, USA) was grown in Dulbecco’s Modified Eagle’s Medium (DMEM) supplemented with 10% fetal bovine serum, 1 mM L-glutamine and 1 mM penicillin-streptomycin solution. Cells were grown in an incubator at 37 °C in a humidified atmosphere (95% air and 5% of carbon dioxide).

### Cellular antioxidant activity assay

Endothelial cells EA.hy926 were seeded on 96-well plates (10^4^ cells/well), containing 100 μL of complete DMEM/well and grown for 24 hours. Then, the cells were washed twice with PBS, and incubated for 30 minutes with bilirubin or biliverdin, dissolved in 100 μL MGD solution (incomplete albumin-free DMEM, 1 mM L-glutamine, 50 μM DCFH-DA) at the following concentrations: 0 (control), 0.5, 2, 5, 10, 25, 50, 75 and 100 nM. After incubation, the solution was removed and the cells were washed twice with PBS. Then, the cells were treated with the peroxyl radical-generating reagent ABAP, at 5 mM in 100 μL Hank’s buffered saline solution (HBSS). The blank wells were filled with HBSS solution without ABAP. The fluorescence was measured at 535 nm with excitation at 485 nm every 5 min for one hour at 37 °C on a microplate reader (Synergy™ H1, Bio-Tek Instruments, Winooski, VT, USA). To quantify the cellular antioxidant activity, the CAA units (%) were calculated as follows:


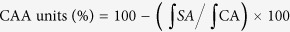


where ∫SA is the integrated area under the sample fluorescence readings (with the blank subtracted) versus the time curve, and ∫CA is the integrated area under the control curve.

### Bilirubin uptake study

Endothelial cells (Ea.hy926) were grown in flasks (75 cm^2^) in completed DMEM for 48 hours to reach confluence. Monolayers were washed with PBS twice. Then, 500 μL of 50 nM bilirubin solution (dissolved in PBS) were added for 30 minutes. All experiments were performed in the dark room.

### Heme oxygenase-1 (HO-1) induction

Endothelial cells (Ea.hy926) were grown in flasks (75 cm^2^) in completed DMEM for 24 hours. Then, they were incubated in CoPPIX solution (10 μM) dissolved in fresh completed DMEM for 24 hours before harvesting. CoPPIX is widely applied as a HO-1 inducer in various experimental models, especially *in vitro* cellular studies, where 3 μM CoPPIX induced maximal level of HO-1 expression at 18 hours of incubation^6^.

### Cell harvesting and sample preparation

Cells were washed with PBS twice, scraped in 500 μL of MeOH: DMSO (1:1, *v*/*v*) and collected into an Eppendorf vial. This protocol was repeated twice. The pooled harvest was ultrasonified for 1 min in an ice bath, rewarmed to room temperature and centrifuged at 12.000 rpm. To determine total protein concentration in the sample, we used Bradford assay on 20 μL of supernatant. The rest of supernatant was diluted with deionized water (1:1, *v*/*v*) to make the final sample prior to HPLC-TLS, HPLC-DAD or HPLC-ESI-MS analysis. In order to improve signal-to-noise ratio due to bilirubin’s low ionization efficiency and its low concentration in the 10 mL-injection loop of the UPLC system, some samples were concentrated by liquid-liquid extraction using chloroform (0.5 mL) prior to mass spectrometric analysis. The bottom chloroform phase was decanted to a new Eppendorf vial and the solvent was removed by a stream of nitrogen gas. The residue was redissolved in DMSO:H_2_O (1:1, v/v) to obtain a final volume of 300 μL (concentrated bilirubin solution).

### Bilirubin analysis in cell samples

All mass spectrometry data were obtained by Waters Acquity ultra-performance liquid chromatograph (UPLC, Waters Corp., Milford, MA, USA) using the flow rate of 0.7 mL/min, the injection volume of 10 μL and the BEH C18 column (1,7 μm, 50 × 2,1 mm i.d.). Other chromatographic conditions for HPLC-TLS method were as previously published^1^.

The LC system was coupled to a hybrid quadrupole orthogonal acceleration time-of-flight mass spectrometer (Q-ToF Premier, Waters, Milford, MA, USA); analyses were made in negative ion mode using electrospray chemical ionization (ESI). The capillary voltage was 3.0 kV, while the sampling cone voltage was 20 V. The source and desolvation temperatures were 130 °C and 400 °C, respectively. The flow rate of nitrogen desolvation gas was 500 L/h. The acquisition range was between *m*/*z* 50 and *m*/*z* 1000 with argon serving as a collision gas at a pressure of 4.5 × 10^−3^ mbar in the T-wave collision cell. The tandem mass spectrometric analysis (MS-MS) was performed using collision energies from 15 to 30 eV to generate the product ion spectra that provided the best structural information. The data were collected in centroid mode, with a scan accumulation time of 0.2 s and an interscan delay of 0.025 s. The data station utilized the MassLynx v4.1 operating software. Accurate mass measurements (Supplementary Information Figure S2) were obtained with an electrospray dual sprayer using the reference compound leucine enkephalin ([M + H^+^]^+^ = 556.2271) at a high mass resolution of approximately 10000 full width of the peak at half its maximum height (FWHM) UPLC-MS spectrum in the Fig. 2c was obtained after a sample (from the control group) 5-fold pre-concentration (by solvent evaporation under reduced pressure), thus in the 3–6 min time range some peaks appear due to bilirubin degradation during sample concentration. Sample concentration prior analysis was needed, since the bilirubin signal intensity (*m*/*z* of 583,2) in the analysed sample (from the control group) was almost insignificant (under LOD), as shown in Supplementary Information (Figure S3).

Limit of detection (LOD) and limit of quantification (LOQ) were determined using the peak representing blirubin based on a visual determination of a peak-to-peak signal-to-noise ratio of at least 3:1 and 10:1, respectively.

### Animals

Adult male Wistar rats weighing 330–350 g (5 months of age) were housed under standard laboratory conditions in a temperature-controlled environment (22 ± 1 °C, 60% humidity) maintained on a 12-h light/dark cycle. All animal procedures and study protocols were approved and conducted in accordance with the permission issued by the Veterinary Administration of the Republic of Slovenia (permit SI-No. U34401-4/2014/4, given to Institute of Pharmacology, Faculty of Medicine, University of Ljubljana), which conforms with the Guide for the Care and Use of Laboratory Animals published by the US National Institutes of Health (NIH Publication No. 85-23, revised 1996).

### Blood vessel isolation

Three rats were killed by cervical dislocation and bled. From each animal both aorta and vena cava inferior were dissected from the thorax, cleaned of connective tissue and periadventitial fat. To remove any remaining blood, blood vessels were rinsed with cold PBS three times (by using a syringe), and were left for additional 5 minutes in the 50 mL beaker filled with the cold PBS solution under dark conditions. To expose the complete endothelium surface, all blood vessels were carefully opened by a longitudinal cut. The endothelial cells were gathered by gentle abrasion with a clean scalpel blade from the edges to the center of the blood vessel. Detached cells were immediately collected in 100 μL of MeOH: DMSO (1:1, v/v) into an Eppendorf vial. The pooled harvest was ultrasonified for 1 min in an ice bath, rewarmed to room temperature and centrifuged at 12.000 rpm. To determine total protein concentration in the sample, we used Bradford assay on 20 μL of supernatant. The rest of supernatant was diluted with deionized water (1:1, v/v) to make the final sample prior to HPLC-TLS.

## Additional Information

**How to cite this article**: Ziberna, L. *et al*. Bilirubin is an Endogenous Antioxidant in Human Vascular Endothelial Cells. *Sci. Rep*. **6**, 29240; doi: 10.1038/srep29240 (2016).

## Supplementary Material

Supplementary Information
