# Supplementary material for: Bilirubin is an Endogenous Antioxidant in Human Vascular Endothelial Cells
Source: Sci Rep. 2016 Jul 6;6:29240. doi: 10.1038/srep29240 (PMC4933905; doi:10.1038/srep29240)
Supplement: Supplementary Information [file srep29240-s1.pdf]

Supplementary Information

BILIRUBIN IS AN ENDOGENOUS ANTIOXIDANT IN HUMAN VASCULAR ENDOTHELIAL CELLS

Lovro Ziberna[1], Mitja Martelanc[2], Mladen Franko[2], Sabina Passamonti[1,*]

[1] *University of Trieste, Department of Life Science, via L. Giorgeri 1, IT-34127 Trieste, Italy*

[2] *University of Nova Gorica, The Laboratory for Environmental Research, Vipavska 13, POB 301, SI-5001 Nova Gorica, Slovenia*

1. HPLC-DAD analysis

Experimental data from HPLC-DAD analysis are described in our previous publication.[1]

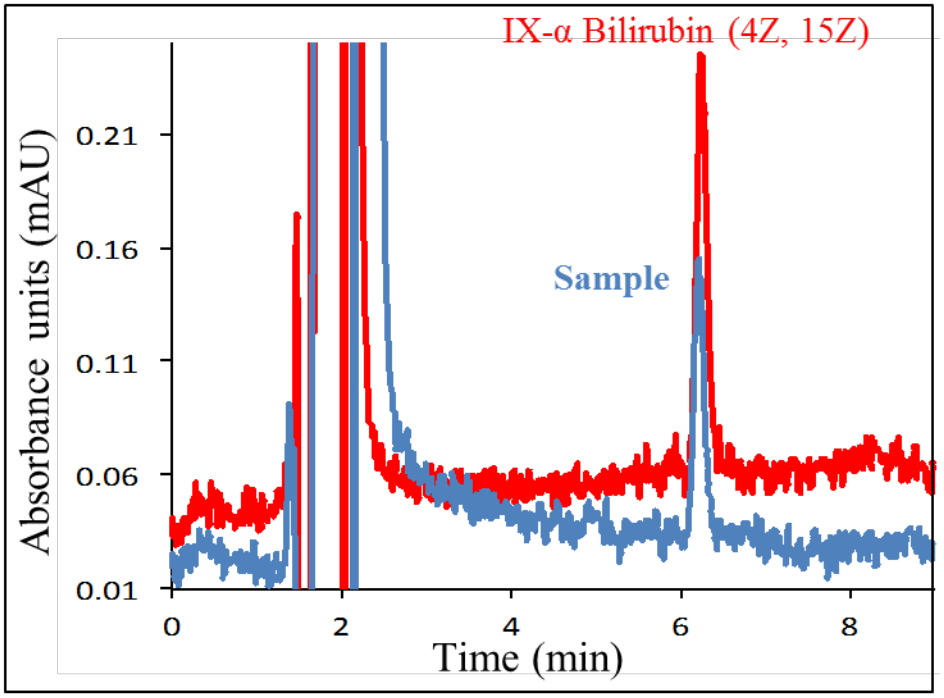

Supplementary Figure S1. HPLC-DAD Analysis. HPLC-DAD chromatograms of bilirubin standard (10nM, red line) and endothelial cellular fraction (blue line). Co-elution of both HPLC peaks additionally proved the presence of IXα bilirubin in the sample. Comparison between absorbance spectra (using peak purity function by the DAD detector), which were taken in the

area across a chromatographic peak of analyzed bilirubin standard solution and the peak present in the sample from the control group is shown in Figure 2b.

Limit of detection (LOD) and limit of quantification (LOQ) were determined using the peak representing bilirubin based on a visual determination of a peak-to-peak signal-to-noise ratio of at least 3:1 and 10:1, respectively. The detector coupled to the HPLC system, i.e. either one based on TLS or a DAD, determines the detection and quantification performance of the methods, as detailed in Supplementary Table 1.

2. High Resolution Mass Spectrometry (HRMS) analysis of bilirubin in a sample of endothelial cells.

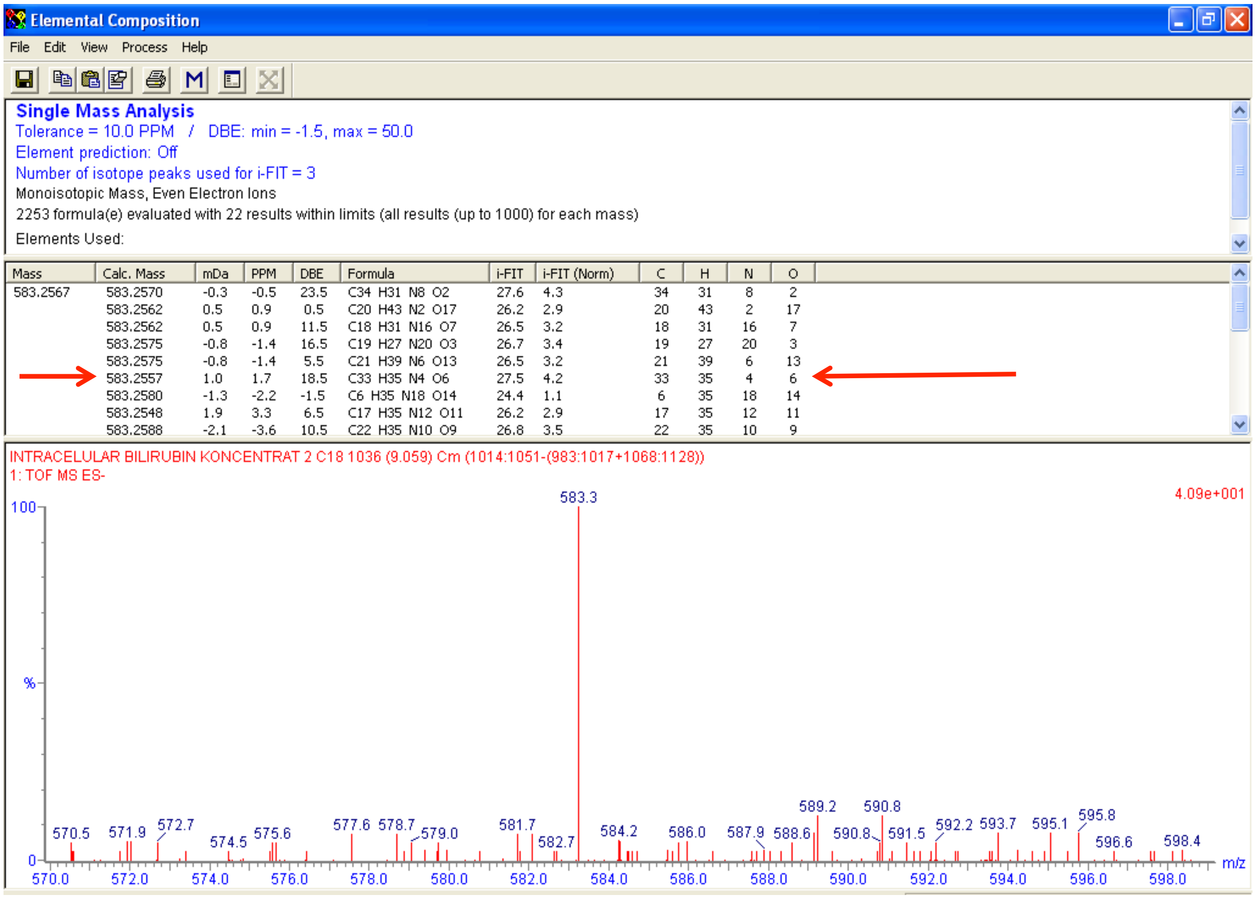

Mass	Calc. Mass	mDa	PPM	DBE	Formula	i-FIT	i-FIT (Norm)	C	H	N	O
583.2567	583.2570	-0.3	-0.5	23.5	C34 H31 N8 O2	27.6	4.3	34	31	8	2
	583.2562	0.5	0.9	0.5	C20 H43 N2 O17	26.2	2.9	20	43	2	17
	583.2562	0.5	0.9	11.5	C18 H31 N16 O7	26.5	3.2	18	31	16	7
	583.2575	-0.8	-1.4	16.5	C19 H27 N20 O3	26.7	3.4	19	27	20	3
	583.2575	-0.8	-1.4	5.5	C21 H39 N6 O13	26.5	3.2	21	39	6	13
	583.2557	1.0	1.7	18.5	C33 H35 N4 O6	27.5	4.2	33	35	4	6
	583.2580	-1.3	-2.2	-1.5	C6 H35 N18 O14	24.4	1.1	6	35	18	14
	583.2548	1.9	3.3	6.5	C17 H35 N12 O11	26.2	2.9	17	35	12	11
	583.2588	-2.1	-3.6	10.5	C22 H35 N10 O9	26.8	3.5	22	35	10	9

Supplementary Figure S2. High Resolution Mass Spectrometry (HRMS) Analysis. Print screen of HRMS results obtained from exact mass analysis of *m/z* signal of 583.3 shown in mass spectrum. The signal was obtained from the analysis of the sample of endothelial cells (from the

control group) in the range of the chromatographic peak representing bilirubin as shown in Figure 2a and 2b (blue chromatographic line). Two red arrows show the correct elemental composition of bilirubin's molecular ion (in negative mode).

3. UPLC-MS analysis of a sample of endothelial cells without pre-concentration

The bilirubin signal intensity (*m*/*z* of 583.3) in the analyzed sample (from the control group) was almost insignificant (under LOD) as shown in Supplementary Figure S1.

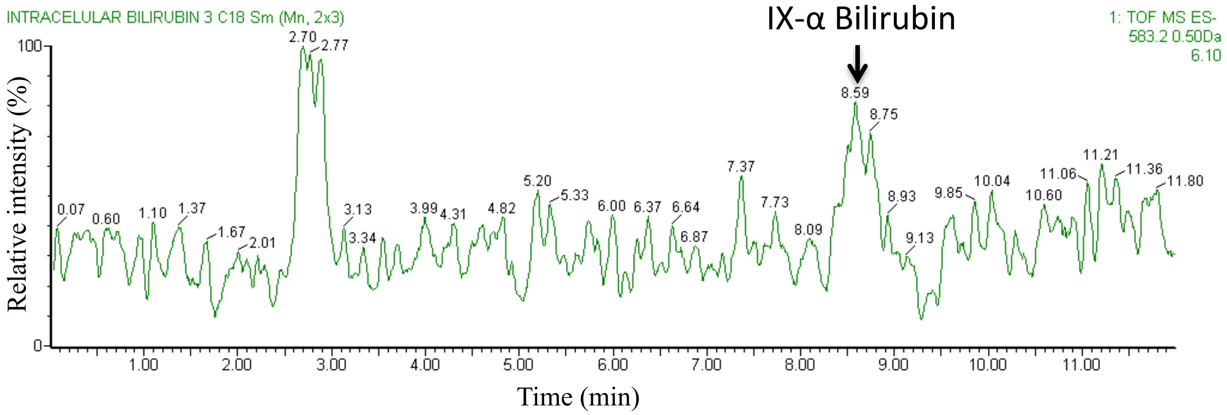

Supplementary Figure S3. UPLC-MS analysis of the sample (from the control group) without pre-concentration, using the flow rate of 0.5 mL/min in order to increase the ionization yield in ESI source due to lower amount of mobile phase that passes through the ion source. No peak detection was observed without pre-concentration. The signal marked with black arrow corresponds to *m*/*z* value of 583.2 (+/- 0.2).

Supplementary Table 1. Limit of detection (LOD) and Limit of quantification (LOQ) of bilirubin in the cellular extract.

	pmol mg^{-1} protein
LOD (HPLC-TLS)	0.04
LOD (HPLC-DAD)	0.80
LOQ (HPLC-TLS)	0.12
LOQ (HPLC-DAD)	3.50

REFERENCE

1. Martelanc, M., Ziberna, L., Passamonti, S. & Franko, M. Direct determination of free bilirubin in serum at sub-nanomolar levels. *Analytica chimica acta* **809**, 174-182 (2014).
